# Quality of life outcomes in Brazilian adults with childhood-onset growth hormone deficiency: impact of transition-age growth hormone therapy

**DOI:** 10.20945/2359-4292-2026-0105

**Published:** 2026-09-12

**Authors:** Kettyuscia Coelho e Oliveira, Mariana Peduti Halah, Luís Eduardo Cruvinel Pinto, Thaís Milioni Luciano, Ryann Luccas de M. Souza, Renata D. Sicchieri Pugliesi, Davi C. Aragon, Paula C. L. Elias, Margaret de Castro, Sonir R. Antonini

**Affiliations:** 1 Universidade do Estado do Amazonas, Faculdade de Medicina, Fundação Hospital Adriano Jorge, Departamento de Clínica Médica, Manaus, AM, Brasil; 2 Departamento de Pediatria, Faculdade de Medicina de Ribeirão Preto, Universidade de São Paulo, Ribeirão Preto, SP, Brasil; 3 Departamento de Clínica Médica, Faculdade de Medicina de Ribeirão Preto, Universidade de São Paulo, Ribeirão Preto, SP, Brasil

**Keywords:** GHD, GHRT, QoL-AGHDA, QoL

## Abstract

**Objective:**

This study evaluated the QoL of Brazilian adult patients with childhood-onset growth hormone deficiency GHD (CO-GHD), comparing those who continued GH replacement therapy (GHRT) after transition to adulthood with those who discontinued treatment and with healthy subjects.

**Subjects and methods:**

In this cross-sectional study, quality of life (QoL) was assessed using the Quality of Life Assessment of Growth Hormone Deficiency in Adults (QoL-AGHDA) questionnaire in 61 Brazilian adults aged 18-50 years. The sample included 31 patients with CO-GHD confirmed during the transition period, who continued or discontinued GHRT, and 30 matched controls.

**Results:**

GHD patients had poorer QoL, as indicated by higher QoL-AGHDA scores than controls (median 8 [IQR 5-14] vs 4 [IQR 2-8]; *p* < 0.01). Continued GHRT in adulthood did not impact QoL compared with discontinued GHRT (8 [IQR 6-16] vs. 9 [IQR 5-12], *p* = 0.33). Overall, women had lower QoL irrespective of GHD (9 [IQR 6-13] vs. 5 [IQR 2-8.5], *p* = 0.01, women and men, respectively). We found no association between QoL-AGHDA scores and education level (*p* = 0.51), short stature (*p* = 0.78), associated pituitary deficiencies (*p* = 0.34), or overweight/obesity (*p* = 0.16).

**Conclusion:**

GHD impairs quality of life in young adults; however, our cross-sectional cohort did not show significant improvements in quality of life associated with GH replacement therapy. A multidisciplinary approach combining individualized treatment and psychosocial interventions remains essential.

## INTRODUCTION

The clinical presentation of hypopituitarism spans from isolated hormonal deficiency to complete absence of pituitary hormone production. Concomitant central nervous system abnormalities can be present in some cases (^1^,^2^). Growth hormone deficiency (GHD) is the most common isolated pituitary hormone deficiency (^3^,^4^). GHD leads to changes in body composition: higher body fat percentage and central obesity; lower lean mass and bone density; and higher risk for fracture. Patients with GHD have higher cardiovascular risk, higher total cholesterol, triglycerides, and low-density lipoproteins. Adults with GHD also report reduced physical capacity and decreased quality of life (QoL). These clinical manifestations vary with chronological age, age of onset of GHD, and other concomitant multiple pituitary hormone deficiencies (MPHD) (^5^-^8^).

QoL scores are important tools in research, clinical practice, and global health care policies (^9^). Compromised QoL is a main concern in adult patients with GHD, with concentration, memory, and tiredness being the most affected parameters (^9^-^11^). The Assessment of Quality of Life in Adults with Growth Hormone Deficiency (QoL-AGHDA) questionnaire is a specific tool and the most used questionnaire for GHD patients (^12^,^13^). It evaluates memory, concentration, fatigue, tension, social isolation, and self-confidence. QoL evaluation enables a cost-benefit analysis, which may help to determine the need for prolonged treatment with GH from childhood to adult life (^14^).

GH replacement therapy (GHRT) in adults with GHD improves body composition and bone density (^5^,^15^). However, the impact of GHRT on QoL remains controversial. Some studies demonstrate benefits in QoL in patients who received GHRT (^9^,^11^,^16^), while others show no improvements (^17^).

In Brazil, guidelines indicate which patients should receive GHRT (^18^). However, QoL is currently not part of the decision process. The establishment of QoL scores as validated tools to guide the management of patients with GHD may benefit clinical practice (^19^). The main goal of this study was to evaluate QoL in adult patients with childhood-onset GHD (CO-GHD) who received GHRT during longitudinal growth and continued treatment in adulthood, compared with those who discontinued GHRT after the transition to adult life, as well as in healthy controls. To our knowledge, this is the first study to compare the QoL of adult Brazilian GHD patients with and without GHRT, using a GHD-specific QoL score validated in the country.

## SUBJECTS AND METHODS

### Subjects

This is a cross-sectional study. The inclusion criteria were patients aged 18 to 50 years with CO-GHD, with isolated GHD or multiple pituitary hormone deficiencies (MPHD), who received GHRT for at least 3 years before the start of the study, and who had confirmed GHD after retesting during the transition period. The exclusion criteria were patients who presented with previous functioning pituitary tumors, malignant neoplasia, active inflammatory diseases, psychiatric illnesses, cognitive disabilities, diabetes mellitus, advanced kidney or liver failure, Turner Syndrome and other chromosomal anomalies, physical disabilities that prevented anthropometric measurements, and pregnancy.

GHD was confirmed by insulin tolerance test (ITT) with a cutoff of 5 µg/L in the transition period, and 3 µg/L in adulthood, or by glucagon stimulation test (GST), with a cutoff of 3 µg/L in eutrophic patients and 1 µg/L in overweight or obese individuals (^5^,^20^,^21^). In patients with MPHD and in those with genetic structural abnormalities of the hypothalamus or the pituitary gland, retesting was not necessary (^20^,^21^). MPHD was confirmed by hormone measurements, and adequate treatment was confirmed by normalization of values in the 6 months before the beginning of the study.

We evaluated 61 Brazilian adult patients aged 18 to 50 years. Of these, 31 were diagnosed with CO-GHD, including 15 patients who were undergoing GHRT treatment at the time of the study, 16 patients who had discontinued GHRT use more than two years before the study, and 30 controls matched for age, sex, and body mass index (BMI). This sample of patients represents the total population of eligible patients available at the tertiary center during the study period, between January 2024 and December 2025, who accepted to participate in this study, and 30 controls matched for age, sex, and body mass index (BMI). Therefore, a formal a priori sample size calculation was not performed. Thus, after adjusting the beta-binomial regression model, considering the main objective (comparison between GHRT-C and GHRT-I, we calculated the difference between means, already adjusted for BMI and GH use time, as well as the 95% confidence interval and the effect size, proposed by Cohen, adjusted for the same covariates, resulting from the same model. The difference between the adjusted means is 2.08 (95% CI: -2.94; 7.07), and the effect size (Cohen’s d) is 0.34, indicating a small to medium effect. Among the 31 patients with CO-GHD had isolated GHD (n = 9) or MPHD (n = 22). Patients with MPHD were on replacement therapy with glucocorticoids (prednisone at doses ranging from 2.5 mg to 5 mg per day), thyroxin, gonadal steroids, and desmopressin. Patients in GHRT were using a fixed dose of GH of 0.3 mg/day. The criteria for maintaining or discontinuing growth hormone (GH) therapy during the transition period were not defined for this research protocol. The decision to maintain or discontinue GH therapy during the transition period and into adulthood was guided by the patient’s own preference to continue GH replacement after reaching final height.

Anthropometric measures were evaluated using WHO growth curves, and short stature was defined as a Z-score of 2 standard deviation units or lower. Patients were followed in the Pediatric Endocrinology outpatient clinic until reaching 18-20 years of age and are now followed in the adult outpatient neuroendocrinology clinics at the São Paulo University Hospital of the Ribeirão Preto Medical School (HC-FMRP-USP), a tertiary referral center. Control patients were recruited among the families of patients and Hospital employees.

All patients signed an informed consent form, and the study was approved by the Clinics Hospital Research Ethics Committee (Approval number: CAAE 75126423.0.0000.5440).

### Assessment of QoL

The QoL-AGHDA questionnaire was the instrument used in this study, after a license was obtained from Galen Research. QoL-AGHDA is a 25-question questionnaire scored as 1/0 (yes/no). The total score is calculated for all items, spanning from 0 to 25. Higher scores are associated with worse QoL (^13^); thus, in our study, a score of 11 or higher was considered poor QoL (^22^). The absence of 25 complete answers excluded the patient from the analyses. To compare scores across different subareas of the original questionnaire, we used the five areas defined by Koltowska (^23^), and the results are presented as the proportion of positive responses within each subarea. The questionnaire was administered during interviews supervised by a single author (KCO).

### Anthropometric measurements

Anthropometric measurements were performed in all patients in the morning after a 12-hour fast. Body weight (kg) and height (m) were measured in a digital scale (Welmy W200A). BMI (kg/m^2^) was calculated, and patients were divided among groups: underweight (BMI < 18.5 kg/m^2^), eutrophic (≥ 18.5 and < 25 kg/m^2^), overweight (≥ 25 an < 30 kg/m^2^), and obese (≥ 30 kg/m^2^). Abdominal circumference was measured with a flexible and non-elastic metrical tape at the midpoint between the inferior costal margin and the iliac crest.

### Statistical analysis

All numeric variables were examined for distribution using histograms, boxplots, and QQ-plots. The most appropriate central tendency measure was selected based on the distribution, and values are presented as means (± standard deviation, SD) or median (min-max or interquartile range [IQR]). Frequency tables for nominal categorical variables were compared using Fisher’s Exact Test or Pearson’s x^2^ test. Proportions for binary variables were compared among ordinal categories using an ordinal logistic regression (proportional odds model). Normally distributed variables were compared between groups using the Student’s t-test. Nonparametric variables were compared using the Mann-Whitney U Test. Pearson correlation was used to test correlations between numeric variables. To compare QoL-AGHDA scores among groups, we fitted a beta-binomial regression adjusted for sex, education level, sedentarism, alcoholism, presence of associated pituitary deficiencies, short stature, and obesity. All analyses and figures were made using open-source packages for R (v4.5.2): glmmTMB v1.1.14; emmeans v2.0.1 or Python (v3.13): matplotlib v3.10.8, numpy v2.4.2, pandas v3.0.1, scipy v1.17.0, seaborn v0.13.2, stats models v0.14.6.

## RESULTS

The final study sample consisted of 31 patients with GHD and 30 controls, most of whom were males (^22^; ^21^, respectively), aged 18-47 and 19-49, respectively. The individual characteristics of each study participant are described in Supplementary Table 1. All of them completed the questionnaire in less than five minutes with no missing answers. Anthropometric, clinical, social, and demographic characteristics of the participants are described in Table 1. There were no differences in age, sex distribution, BMI, or marital status between GHD patients and controls. Patients with GHD were shorter, had a higher proportion of sedentary, and a lower proportion of alcohol consumption than controls. Moreover, the distributions of employment status and education levels differed between the groups, and controls were more likely to have completed 13 or more years of education. One patient from the control group was diagnosed with high blood pressure.

**Table 1.** Anthropometric and demographic characteristics of the participants

	GHD (n = 31)	Controls (n = 30)	*p*
Age (years)	26 (18 – 48)	27 (20 – 49)	0.68
Males (n)	22 (71%)	21 (70%)	1.0
Height (SDS)	-1.3 (-3.3 – 0.7)	-0.3 (-1.9 – 3.0)	<0.01
BMI (kg/m²)	24.4 (15.4 – 39.3)	23.1 (17.7 – 39.4)	0.92
Overweight or obesity (n)	14 (45.2%)	14 (46.7%)	1.0
Smokers (n)	1 (3.2%)	3 (10%)	0.35
Alcoholism (n)	5 (16.1%)	13 (43.3%)	0.03
Sedentary (n)	23 (74.2%)	8 (26.7%)	<0.01
Marital status			0.11
Married	7 (22.6%)	13 (43.3%)	
Single	24 (77.4%)	17 (56.7%)	
Employment status			<0.01
Unemployed	8 (25.8%)	0	
Student	5 (16.1%)	15 (50%)	
Working	18 (58.1%)	15 (50%)	
Education level			<0.01
5 years or less	3 (9.7%)	1 (3.3%)	
6 – 9 years	1 (3.2%)	0	
10 – 12 years	20 (64.5%)	3 (10%)	
13 years or more	7 (22.6%)	26 (86.7%)	

GHD: growth hormone deficiency; BMI: body mass index; SDS: standard deviation score. Data are presented as medians (min – max), or absolute counts (proportion).

The clinical characteristics of participants with GHD are described in Table 2 and are divided between those who stopped and those who continued receiving GHRT after the transition period. The median time between interruption of treatment from the transitional period and the evaluation was 6.1 years (SD ± 2.4). Participants who interrupted GHRT had a lower BMI and a lower total duration of treatment since childhood. The distribution of GHD etiologies across subgroups was similar, as were the proportions of multiple pituitary deficiencies (Table 2). Most patients had congenital idiopathic GHD, and more than two-thirds had at least one other pituitary hormone deficiency.

**Table 2.** Clinical characteristics, distribution etiologies and distribution of associated pituitary deficiencies in the patients with growth hormone deficiency divided by treatment interruption or maintenance during the transition period

	GHRT-C (n = 15)	GHRT-I (n = 16)	*p*
Males (n)	11 (73%)	11 (69%)	1.0
Age at diagnosis (years)	8 [6.5; 9.5]	12 [8; 14]	0.11
BMI (kg/m²)	27.6 [22.4; 33.1]	21.7 [19.6; 25]	0.03
Height (SDS)	-0.6 (-2.3; 0.5)	-1.5 (-3.3; 0.7)	0.11
Total treatment (years)	17 (12 - 30)	6 (3 – 20)	< 0.01
GHD etiology			1.0
Congenital GHD	12 (80%)	13 (81%)	
Craniopharyngioma^§^	1 (6.7%)	2 (12.5%)	
Non-functional pituitary adenoma	1 (6.7%)	0	
Langerhans cell histiocytosis	0	1 (6.2%)	
Astrocytoma	1 (6.7%)	0	
Other pituitary deficiencies			
None	4 (26.7%)	5 (31.2%)	1.0
TSH	11 (73,3%)	11 (68.8%)	1.0
AVP	2 (13.3%)	4 (25%)	0.65
ACTH	10 (66,7%)	8 (50%)	0.47
LH/FSH	10 (66.7%)	7 (43.8%)	0.29

Data are presented as medians (min-max), median [interquartile range, IQR] or absolute counts (proportions, %).

§ Two patients received cranial irradiation. GHRT-C: GHD patients who continued growth hormone replacement therapy in the transition period; GHRT-I: GHD patients who interrupted growth hormone replacement therapy in the transition period; BMI: body mass index; SDS: standard deviation score; GHD: growth hormone deficiency; TSH: thyroid-stimulating hormone; AVP: arginine vasopressin; ACTH: adrenocorticotropic hormone; LH: luteinizing hormone; FSH: follicle-stimulating hormone.

**Figure 1:**
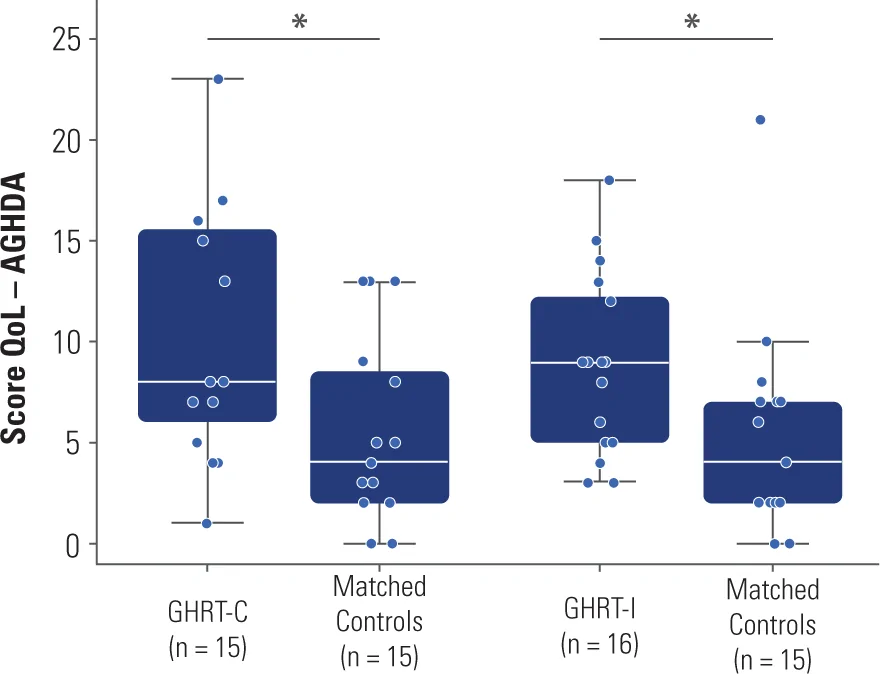
Distribution of QoL-AGHDA scores for each group

QoL-AGHDA: quality of life assessment of growth hormone deficiency (GHD) in adults; GHRT-C: GHD patients who continued growth hormone replacement therapy in the transition period; GHRT-I: GHD patients who interrupted growth hormone replacement therapy in the transition period. *p < 0.05.

The QoL-AGHDA scores of the patients with GHD were higher than the scores of matched controls (median 8 [IQR 5 – 14] vs 4 [IQR 2 – 8]; *p* < 0.01), adjusted by sex, education level, sedentary lifestyle, alcoholism, smoking, presence of other pituitary deficiencies, short stature, and obesity. There were no differences between participants using GHRT and those who interrupted GHRT (8 [IQR 6 – 16] vs. 9 [IQR 5 – 12], respectively, *p* = 0.33). These results are shown in Figure 1.

**Figure 2:**
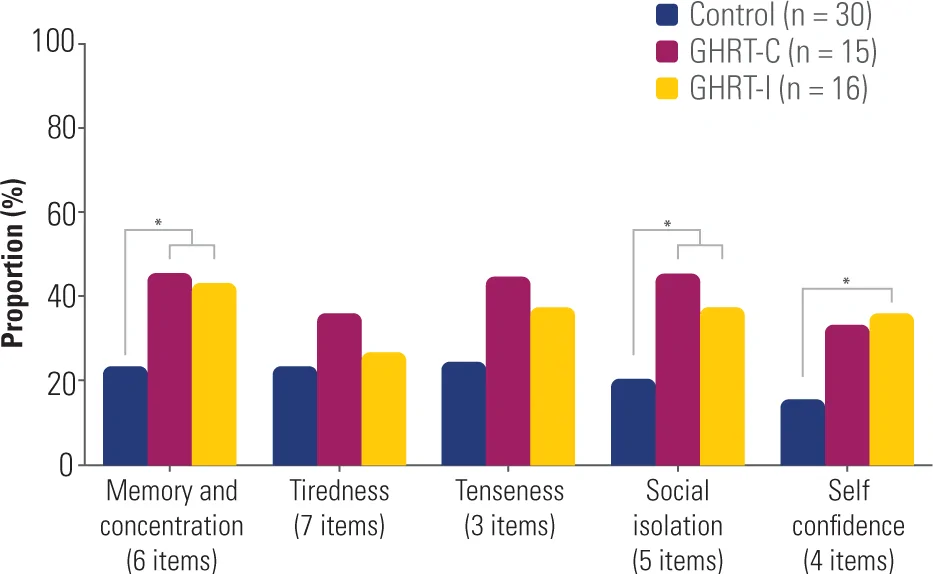
Average proportion of positive^†^ answers for each group across subareas of the QoL-AGHDA questionnaire

QoL-AGHDA: quality of life assessment of GHD in adults; GHRT-C: GHD patients who continued growth hormone replacement therapy in the transition period; GHRT-I: GHD patients who interrupted growth hormone replacement therapy in the transition period.

^†^positive represents the presence of such a parameter or an impaired parameter; *p < 0,05.

There were no predominant sub-areas across the three groups, except for the patients who discontinued GHRT, who presented higher scores in memory and concentration than in fatigue (*p* = 0.02). These results are shown in Figure 2. In the analysis of each specific domain of the QoL-AGHDA, it was observed that both groups with GHD showed higher proportions of positive responses than the controls for memory and concentration (*p* = 0.02 for both), but no difference was found between the groups with GH deficiency (*p* = 0.85). Regarding social isolation, there was a difference between the continued GHRT group vs Control (*p* = 0.01) and between the discontinued GHRT group vs. Control (*p* = 0.03). In terms of self-confidence, the discontinued GHRT group had higher scores than controls (*p* = 0.02). The distribution of positive answers for tiredness and tenseness did not differ among patients in the continued GHRT, discontinued GHRT, and control groups (Figure 2).

**Figure 3:**
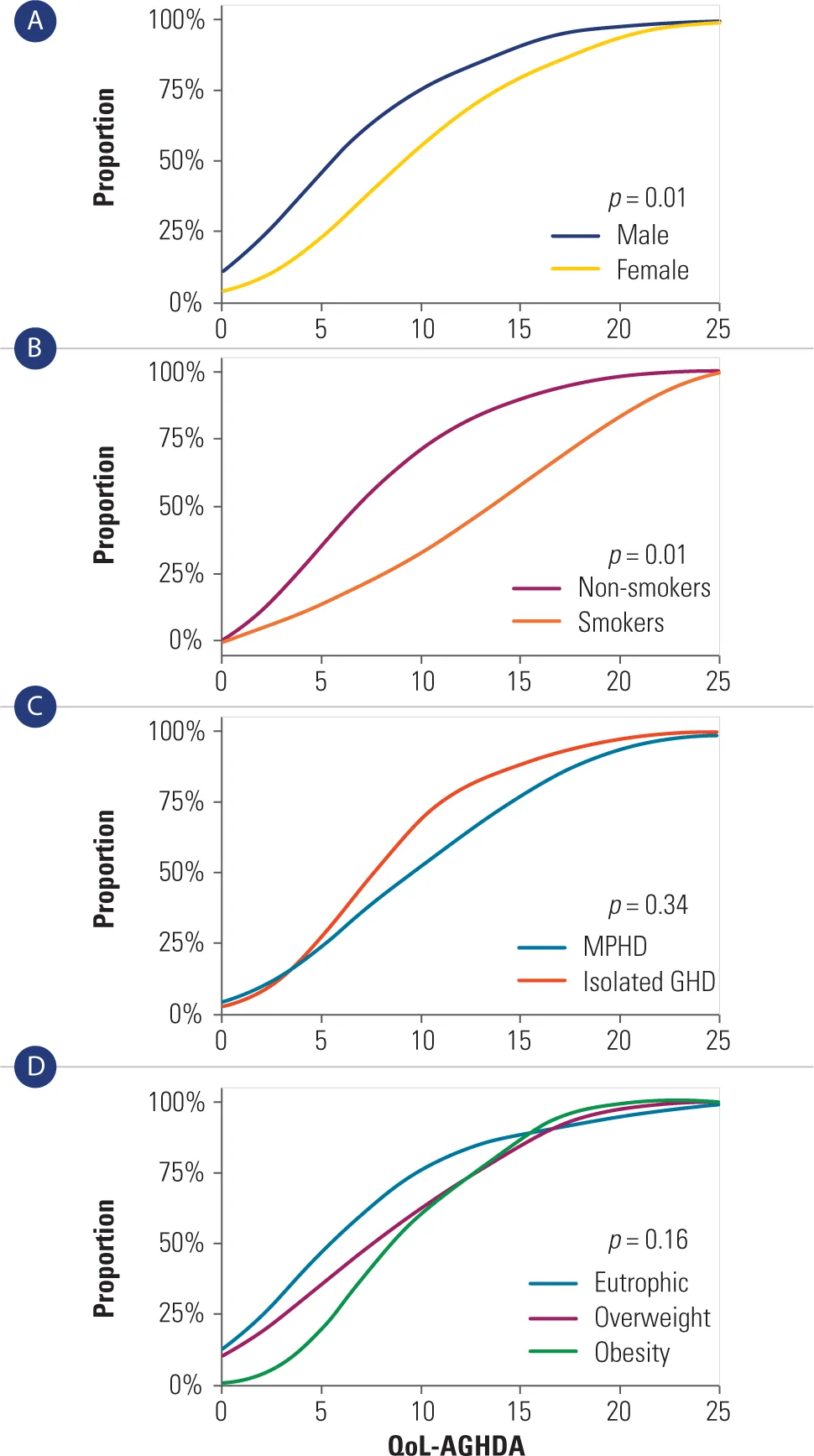
Cumulative distribution of QoL-AGHDA scores according to group, gender, smoking, presence of associated pituitary deficiencies, or BMI category. Cumulative distribution of QoL-AGHDA scores by: (A) gender (n = 61); (B) smoking (n = 61); (C) the presence of associated pituitary deficiencies (n = 31); (D) BMI category (n = 61)

QoL-AGHDA: quality of life assessment of GHD in adult questionnaire; BMI: body mass index; MPHD: multiple pituitary hormone deficiencies; GHD: growth hormone deficiency.

The QoL-AGHDA scores were higher in women than in men (9 [IQR 6 – 13] vs. 5 [IQR 2 – 8.5], *p* = 0.01, Figure 3A), independently of the diagnosis of GHD. Smokers also had higher scores than non-smokers (14 [IQR 10 – 17] vs. 7 [IQR 3 – 9], *p* = 0.01, Figure 3B). We found no association between QoL-AGHDA scores and education level (*p* = 0.51), sedentary lifestyle (*p* = 0.70), alcoholism (*p* = 0.45), short stature (*p* = 0.78), presence of multiple pituitary deficiencies (*p* = 0.34, Figure 3C), or overweight/obesity (*p* = 0.16, Figure 3D). Among patients with GHD, the mean difference in scores between those younger than 30 years and those older than 30 years was -1.40 (95%CI: -5.2; 3.2).

The mean difference in QoL-AGHDA scores between GHD patients with treatment interruption for less than five years and those who had interruption for five years or more was 1.2 (95%IC -2.9; 6.8). There was no correlation between interruption time and QoL-AGHDA scores (*p* = 0.7; r = 0.1).

There was no correlation between QoL-AGHDA scores and BMI (*p* = 0.38; r = 0.11), height SDS (*p* = 0.16; r = -0.18), or IGF-1 levels (*p* = 0.03, r = -0.28). A beta-binomial regression model was fitted, including BMI and treatment duration as covariates. The mean difference was 2.08 (95% CI: -2.94 to 7.07; *p* = 0.41), indicating that even after accounting for potential inter-group discrepancies regarding these covariates, no significant difference in quality of life was observed between the groups.

## DISCUSSION

In the present study, we applied QoL-AGHDA, a Brazilian Portuguese validated instrument (^13^,^19^), to assess QoL in patients with childhood-onset GHD confirmed during the adolescence to adult transition period, who continued or discontinued GHRT and matched controls. Untreated patients typically exhibit poorer QoL, while GHRT improves outcomes toward normative values (^23^). Current guidelines recommend continuation of GHRT until peak somatic development, with restricted use thereafter based on severity and QoL impairment (^22^).

Our study showed that patients with GHD were shorter than controls with no differences in age, sex distribution, BMI, or marital status. Many of these similar characteristics between the groups are important to strengthen the differences, such as GHD patients had a higher proportion of sedentarism, more unemployment status, and were more likely to have fewer years of education, which implicates a putative low QoL. On the other hand, adults with childhood-onset-GHD present lower QoL than controls, which is consistent with previous reports indicating poorer outcomes observed in patients with GHD (^24^,^25^). This finding can be attributable to GHD, besides a multifactorial burden, involving adverse body composition, reduced physical performance, metabolic disturbances, and neuroendocrine alterations, all of which negatively impact cognitive function and psychological well-being (^7^,^11^,^26^). Thus, both disease-specific factors, socioeconomic and genetic determinants, may collectively explain the observed QoL outcome. It has already been recognized that prevalent comorbidities, such as anxiety and depression, are likely involved in the complex interplay of genetic and psychosocial determinants (^4^), alongside GH/IGF-1 axis dysregulation (^27^). Of interest, GHD patients had a lower proportion of alcohol consumption than controls. Studies have suggested better adaptation of childhood-onset GHD patients to their functional limitations compared to adult-onset GHD cohorts, who perceive a more acute decline in QoL (^11^,^13^).

Although GHRT leads to progressive improvement across QoL domains, earlier in social functioning and later in cognitive aspects (^9^,^11^,^16^), our study found no differences between childhood-onset GHD patients who early stopped GHRT at the transitional period (6.1 years before the study) and those who continued receiving GHRT after the adolescence to adult transition period. This may be explained by the young age of the cohort, adaptive mechanisms, and limited statistical power. As expected, patients who interrupted GHRT during the transitional period had a lower total duration of treatment since childhood, whereas both groups presented similar GHD etiologies as well as the proportions of multiple pituitary deficiencies, age at diagnosis, and proportion by gender, which enable us to appropriately compare these groups regarding metabolic parameters and QoL.

While the physiological efficacy of GHRT on body composition, linear growth, and bone mineral density is unequivocally established, the effect of maintenance of GH treatment throughout the adolescence to adult transitional period on QoL remains a subject of debate (^5^,^7^,^15^,^28^). While some authors show that most patients with GHD and other pituitary hormone deficiencies show a long-lasting improvement of QoL after GHRT (^9^,^11^,^16^,^26^), others show no difference in those who received or did not receive GH treatment throughout adolescence to the adult transitional period (^17^).

Our data showed no differences between adult patients on GHRT and those who interrupted GHRT in accordance with previous studies (^4^,^14^). One study also demonstrated that children and adolescents with GHD treated with GH exhibit health-related QoL comparable to that of healthy controls (^29^). Although the overall quality of life may not decline immediately after discontinuation of GHRT upon attainment of adult height, patients with childhood-onset GHD may experience significant age-specific psychosocial impairments, including disturbances in body image. These deficits can be alleviated by continued GH therapy; therefore, GHRT should not be discontinued solely after achieving adult height, to prevent progressive psychosocial distress in adulthood and to preserve optimal body composition (^30^).

Interestingly, the duration of GHRT itself does not seem to significantly influence QoL or mood states, as comparable outcomes have been observed between patients treated for shorter versus longer periods. Consistent with these observations, in our cohort, patients who had received long-term GHRT (mean duration 17.5 years) did not differ in QoL scores from those who had discontinued treatment and had remained off therapy for a mean of 6.1 years. These data are also in agreement with a recent study that showed no significant differences in QoL or mood states between patients treated for less than 5 years and those receiving therapy for 5 years or longer (^4^). The improvement in QoL in patients receiving GHRT usually occurs within the first year of treatment and persists for up to 10 years (^26^).

Favorable responses to GHRT have been associated with the absence of depression, lower BMI, absence of visual impairment, worse baseline QoL, isolated GHD, and childhood-onset GHD (^11^,^26^). In contrast, GH dose and IGF-1 levels do not appear to predict QoL outcomes (^4^,^14^,^26^). Additionally, after discontinuation of GHRT, mood states have been reported to correlate negatively with height and age at treatment cessation and positively with BMI (^4^). However, in our study, IGF-1 levels, BMI, and height were not associated with QoL-AGHDA scores.

The QoL-AGHDA captures key domains affected in GHD, such as cognition, fatigue, self-confidence, tension, and social isolation. GHRT leads to progressive improvement, with earlier gains in less affected domains such as social isolation and later recovery in cognitive domains (^9^,^11^,^23^). Long-term data confirm sustained QoL benefits, with fatigue improving within the first year and memory over subsequent years (^16^).

In our sample, there was no predominant compromised subarea in the GHD patients; this contrasts with previous studies that showed concentration, memory, and tiredness to be the parameters most affected (^9^-^11^). These findings may be attributed to the fact that our cohort is substantially young, and it is plausible that age-related cognitive and physical demands are not yet sufficiently pronounced to demonstrate the protective effects of ongoing GHRT. However, when patients with GHD were compared to matched controls, both GHD groups’ scores were higher than those of controls for memory and concentration and social isolation, while tiredness, tenseness, and self-confidence were not significantly different. Since this was a secondary analysis for which the sample size was not optimized, the sample power for this may not have been sufficient to detect smaller differences.

Overall, in our cohort, QoL was influenced by sex, with women reporting significantly lower QoL than men, while no associations were observed with education level, short stature, presence of additional pituitary deficiencies, or overweight/obesity. Actually, women tend to report poorer QoL than men (^31^). Women using oral estrogen as replacement therapy or for contraceptive purposes are more GH-resistant than men because estrogen attenuates GH action on the liver, the principal site of IGF-1 synthesis, resulting in lower IGF-1 generation (^21^), which could exacerbate the decline in quality of life observed in this subgroup. In the general population, QoL-AGHDA normative scores average 4.6, with slightly higher values in women and older individuals (^12^). In our cohort, patients younger than 30 years reported lower QoL than older individuals, although this difference was not statistically significant. Additionally, childhood-onset hypopituitarism has been associated with adverse socioeconomic outcomes, including lower educational attainment, higher unemployment, and fewer relationships (^25^), findings that are consistent with the higher unemployment and lower education levels observed in our GHD cohort compared with controls.

Compared with a previously reported cohort of untreated Brazilian adults with GHD (mean age 46.3 years, predominantly female; median QoL-AGHDA score 11) (^19^), our patients exhibited lower median QoL-AGHDA scores regardless of GHRT status during transition. However, this comparison should be interpreted cautiously, as our cohort was substantially younger (mean age 27.2 years) and predominantly male (71%). Consistent with normative population data showing poorer QoL among women and older individuals (^12^), these demographic differences likely account for the more favorable QoL observed in our study.

Several limitations should be considered when interpreting these findings. First, the relatively small sample size may limit generalizability to the broader adult GHD population. Second, unreported comorbidities and psychosocial factors may have introduced residual confounding in QoL assessments. Although concomitant pituitary deficiencies were appropriately treated, their potential impact on perceived health cannot be entirely excluded. In addition, the cohort showed a skewed gender distribution, with male predominance. Another important point to consider is that the decision to continue or discontinue GH therapy was based on patient preference and not randomization. This non-randomized approach may introduce selection bias, since patients who did not perceive a significant benefit from treatment may have been more likely to discontinue treatment. However, worse quality of life scores were predicted in the discontinuation group due to this potential bias; however, this expected trend was not observed in our cohort, which showed no significant difference in quality-of-life scores between the groups.

Despite these limitations, this study has important strengths. To our knowledge, it is the first to evaluate QoL in Brazilian adults with childhood-onset GHD receiving GHRT, in comparison with childhood-onset GHD who early stopped GHRT at the transitional period and healthy controls, using a rigorously validated, disease-specific instrument adapted for the local population.

In conclusion, although GHD is known to impair quality of life in young adults, our cross-sectional data did not demonstrate a significant association between GHRT and QoL improvements. Future longitudinal research is required to determine the definitive clinical impact of GH replacement. In the meantime, therapy must remain individualized and combined with psychosocial interventions to maximize patients’ overall health.

## Data Availability

the datasets used and/or analyzed during the current study are available from the corresponding author upon reasonable request.

## SUPPLEMENTARY MATERIAL

**Supplementary Table 1.** Characteristics of all individual participants of the study

	Age (years)	Sex	Etiology	Height (SDS)	Weight (kg)	BMI (kg/m^2^)	Age of diagnosis (years)	IGF-1 (SDS)	Current Treatment	Total GHRT (years)	Score QoL-AGHD
GHD patients who continued growth hormone replacement therapy in the transition period											
Pt 1	42	M	Pituitary hypoplasia	-0.62	82	27.72	12	0.05	P-T4-T-GH	30	4
Pt 2	30	M	Pituitary hypoplasia	0.47	81.9	25.28	8	-1.07	P-T-GH	23	13
Pt 3	26	M	Pituitary hypoplasia	-0.62	111	37.52	8	2.34	GH	18	5
Pt 4	27	M	Pituitary hypoplasia	-0.01	64.8	20.8	10	0.12	P-T4-T-GH	17	23
Pt 5	21	M	Pituitary hypoplasia	-0.21	94	30.69	6	-0.29	T4-GH	15	7
Pt 6	38	M	Mutation GHRH	-1.86	73.2	27.55	8	-0.02	GH	18	8
Pt 7	39	F	Mutation GHRH	-0.79	59	23.63	9	-0.38	GH	18	7
Pt 8	25	M	Craniopharyngioma	0.47	87.5	27.01	7	-1.07	P-T4-T-GH-D	17	17
Pt 9	26	F	Pituitary hypoplasia	-1.55	91	38.87	9	-0.03	P-T4-E/P-GH	17	16
Pt 10	26	M	Pituitary hypoplasia	-0.21	65	21.22	8	0.28	P-T4-T-GH	17	4
Pt 11	25	F	Pituitary hypoplasia	-0.48	72	28.12	6	-2.62	P-T4-E/P-GH	19	17
Pt 12	18	M	Pituitary hypoplasia	-0.2	54	17.63	6	-1.29	GH	12	8
Pt 13	20	M	Idiopathic	-2.27	51.4	20.08	5	-0.14	T-GH	13	1
Pt 14	29	M	Nonfunctioning macroadenoma	-2.27	92	35.94	14	-0.79	P-T4-T-GH	15	15
Pt 15	28	F	Astrocytoma	-2.01	80	35.56	14	-2.18	P-T4-E/P-GH	14	8

GHD patients who interrupted growth hormone replacement therapy in the transition period											
Pt 16	18	F	Pituitary hypoplasia	-1.5	46.5	19.86	9	-1.57	---	7	18
Pt 17	47	M	Pituitary hypoplasia	-2.13	66.3	25.58	14	-2.33	P-T4-D	3	14
Pt 18	19	F	Histiocytosis	-0.48	48.3	18.87	12	-1.44	T4-D	3	4
Pt 19	22	M	Craniopharyngioma	-0.01	69.1	22.18	12	-4.46	P-T4-T-D	4	13
Pt 20	22	F	Pituitary hypoplasia	-2.16	34.1	15.36	12	-3.5	P-T4-E/P	3	9
Pt 21	18	F	Pituitary hypoplasia	-1.51	65	27.77	13	-0.44	---	3	9
Pt 22	20	M	Craniopharyngioma	-1.99	103	39.25	14	-3.32	P-T4-T- D	3	6
Pt 23	35	M	Pituitary hypoplasia	-1.58	43.6	16.01	8	-1.99	P-T4-T	18	9
Pt 24	29	M	Pituitary hypoplasia	0.75	82	24.76	6	-3.18	P-T4-T	17	9
Pt 25	22	M	Pituitary hypoplasia	-2.4	50	19.78	8	-1.1	---	7	3
Pt 26	44	M	Pituitary hypoplasia	-1.72	72	26.77	15	-1.83	P-T4-T	20	5
Pt 27	18	M	Pituitary hypoplasia	-3.33	40	17.31	4	-5.42	P-T4-T	11	8
Pt 28	25	M	Pituitary hypoplasia	-0.21	64	20.9	6	-0.78	---	13	5
Pt 29	24	M	Idiopathic	-1.31	68	24.38	10	-3.17	T4	10	3
Pt 30	29	M	Pituitary hypoplasia	-0.62	63	21.3	16	-1.11	T4	4	15
Pt 31	32	F	Pituitary hypoplasia	-2.32	52	23.74	16	-1.66	---	5	12

Controls											
Pt 32	49	M	---	1.57	82	23.2	---	1.18	---	---	0
Pt 33	29	M	---	-0.07	66	21.31	---	1.62	---	---	2
Pt 34	21	M	---	0.54	74.8	22.96	---	-0.42	---	---	2
Pt 35	27	M	---	1.16	135	39.44	---	-1.73	---	---	5
Pt 36	22	F	---	1.66	60	19.82	---	0.71	---	---	4
Pt 37	21	F	---	-1.55	47	20.08	---	0.32	---	---	6
Pt 38	21	F	---	-0.94	63	25.56	---	0.51	---	---	10
Pt 39	36	F	---	3.03	74.7	22.31	---	0.32	---	---	3
Pt 40	21	M	---	1.16	103	30.09	---	1.82	---	---	5
Pt 41	21	M	---	0.88	70	20.9	---	0.33	---	---	7
Pt 42	23	M	---	-1.44	73	26.49	---	-0.83	---	---	0
Pt 43	29	M	---	0.88	85	25.38	---	0.95	---	---	4
Pt 44	27	M	---	-0.49	77.5	25.89	---	0.7	---	---	2
Pt 45	27	M	---	-0.35	76	25.1	---	-0.08	---	---	8
Pt 46	24	M	---	0.13	66	20.95	---	0.69	---	---	2
Pt 47	36	M	---	-1.03	86.5	30.29	---	-0.67	---	---	13
Pt 48	43	M	---	2.39	103	27.37	---	1.27	---	---	2
Pt 49	23	M	---	-1.86	60	22.58	---	0.78	---	---	0
Pt 50	34	F	---	-0.48	56.5	22.07	---	1.11	---	---	2
Pt 51	24	F	---	-0.79	64	25.64	---	1.02	---	---	13
Pt 52	24	M	---	-1.31	62	22.23	---	2,76	---	---	0
Pt 53	21	M	---	-0.62	56	18.93	---	1,48	---	---	2
Pt 54	47	M	---	1.3	93	26.88	---	0.29	---	---	2
Pt 55	19	M	---	-1.03	59	20.66	---	-0.04	---	---	3
Pt 56	27	M	---	0.47	74	22.84	---	0.67	---	---	7
Pt 57	23	F	---	-1.4	42	17.71	---	2.05	---	---	21
Pt 58	32	F	---	-1.09	88	36.16	---	1.66	---	---	8
Pt 59	26	F	---	-0.18	102	38.87	---	0.54	---	---	9
Pt 60	32	M	---	0.47	107	33.02	---	-0.18	---	---	13
Pt 61	37	M	---	-1.58	52	19.1	--	-0.02	---	---	7

Pt: patient; M: male; F: female; BMI: body mass index; P: prednisone; T4: levothyroxine; E/P: estrogen/progesterone; D: desmopressin; GHRT: growth hormone replacement therapy; QoL-AGHDA: quality of life assessment of GHD in adult questionnaire; SDS: standard deviation score.
